# Enhanced killing of androgen-independent prostate cancer cells using inositol hexakisphosphate in combination with proteasome inhibitors

**DOI:** 10.1038/sj.bjc.6604730

**Published:** 2008-10-21

**Authors:** J-S Diallo, B Betton, N Parent, B Péant, L Lessard, C Le Page, R Bertrand, A-M Mes-Masson, F Saad

**Affiliations:** 1 Centre de recherche du Centre hospitalier de l'Université de Montréal (CR-CHUM) and Institut du cancer de Montréal, 1560 rue Sherbrooke est, Montréal, Québec, H2L4M1, Canada; 2 Département de médecine, Université de Montréal, Montréal, Québec, H3C3J7, Canada; 3 Département d'urologie, Université de Montréal, Montréal, Québec, H3C3J7, Canada

**Keywords:** inositol hexakisphosphate, proteasome inhibitors, prostate cancer, BCL-2 family proteins

## Abstract

Effective treatments for androgen-independent prostate cancer (AIPCa) are lacking. To address this, emerging therapeutics such as proteasome inhibitors are currently undergoing clinical trials. Inositol hexakisphosphate (IP6) is an orally non-toxic phytochemical that exhibits antitumour activity against several types of cancer including PCa. We have previously shown that treatment of PC3 cells with IP6 induces the transcription of a subset of nuclear factor-*κ*B (NF-*κ*B)-responsive and pro-apoptotic BCL-2 family genes. In this study, we report that although NF-*κ*B subunits p50/p65 translocate to the nucleus of PC3 cells in response to IP6, inhibition of NF-*κ*B-mediated transcription using non-degradable inhibitor of *κ*B (I*κ*B)-*α* does not modulate IP6 sensitivity. Treatment with IP6 also leads to increased protein levels of PUMA, BIK/NBK and NOXA between 4 and 8 h of treatment and decreased levels of MCL-1 and BCL-2 after 24 h. Although blocking transcription using actinomycin D does not modulate PC3 cell sensitivity to IP6, inhibition of protein translation using cycloheximide has a significant protective effect. In contrast, blocking proteasome-mediated protein degradation using MG-132 significantly enhances the ability of IP6 to reduce cellular metabolic activity in both PC3 and DU145 AIPCa cell lines. This effect of combined treatment on mitochondrial depolarisation is particularly striking and is also reproduced by another proteasome inhibitor (ALLN). The enhanced effect of combined MG132/IP6 treatment is almost completely inhibited by cycloheximide and correlates with changes in BCL-2 family protein levels. Altogether these results suggest a role for BCL-2 family proteins in mediating the combined effect of IP6 and proteasome inhibitors and warrant further pre-clinical studies for the treatment of AIPCa.

Prostate cancer (PCa) remains one of the leading causes of cancer-related death in North-American men (Jemal *et al*, 2007). Though localised forms of the disease can often be successfully treated by surgery or radiotherapy, a significant proportion of patients having undergone such interventions are at risk of disease recurrence. Androgen deprivation therapy can prolong the life expectancy of these patients; however, androgen-independent (AI) PCa (AIPCa) eventually arises. As currently available treatment options for AIPCa are lacking, there is a growing need for novel therapeutics that can be effective against this advanced stage of the disease.

Inhibitors of the proteasome are showing promise as anticancer agents against several cancers. Although the proteasome harbours active sites for several types of proteolytic activity, these inhibitors generally consist of small synthetic peptides targeting the chymotryptic activity of the proteasome, which is thought to be a rate-limiting catalytic step in proteasome-mediated protein degradation (Lee and Goldberg, 1998). Notably, one proteasome inhibitor (bortezomib) has been recently approved by both the Food and Drug Administration (USA) and the European Agency for Evaluation of Medicinal products for the treatment of recurrent multiple myeloma and is currently undergoing clinical trials for AIPCa (Papandreou and Logothetis, 2004; Papandreou *et al*, 2004; Price and Dreicer, 2004; Zavrski *et al*, 2007).

Some studies have suggested that a major downstream target of proteasome inhibition is nuclear factor-*κ*B (NF-*κ*B), a transcription factor involved in the transcriptional regulation of hundreds of genes implicated in cell proliferation, differentiation and cell survival (Chen and Greene, 2004). Nuclear factor-*κ*B is a dimeric protein composed of homo- or heterodimers of p50, p65 (RelA), p52, RelB and c-Rel subunits. Nuclear factor-*κ*B activity is directly regulated by the action of inhibitor of *κ*B (I*κ*B) proteins (e.g., I*κ*B-*α*, -*β*, -*ε* and p100) and I*κ*B kinases (Hoffmann *et al*, 2002; Karin *et al*, 2002; Buss *et al*, 2004). Inhibitors of *κ*B retain NF-*κ*B in the cell cytoplasm and diminish its transactivation potential, whereas I*κ*B kinases phosphorylate both I*κ*Bs as well as NF-*κ*B itself. Importantly, phosphorylation of I*κ*Bs by IKKs leads to their proteasome-mediated degradation and to the release and nuclear translocation of NF-*κ*B subunits (classically p50/p65). Alternately, in what is referred to as the non-canonical pathway, the proteasome catalyses the processing of p100 into the NF-*κ*B p52 subunit, which may also translocate to the nuclear compartment (Karin and Ben-Neriah, 2000; Karin *et al*, 2002; Chen and Greene, 2004).

More recently, proteasome inhibitors have also been found to upregulate the expression of BCL-2 family proteins. Notably, pro-apoptotic BH3-only proteins such as NOXA, BIK/NBK, BIM and PUMA see their levels increased following treatment with proteasome inhibitors such as MG-132, ALLN, lactacystin and bortezomib (Marshansky *et al*, 2001; Nikrad *et al*, 2005; Zhu *et al*, 2005; Qin *et al*, 2006; Concannon *et al*, 2007). In parallel, antiapoptotic proteins such as MCL-1 can also be upregulated following proteasome inhibition, leading investigators to combine proteasome inhibitors with strategies aimed at thwarting the antiapoptotic response with some success (Qin *et al*, 2006).

BCL-2 family proteins play a major role in the control of mitochondrial permeability. Importantly, mitochondrial outer-membrane permeation (MOMP) is a key event in cell death whether by means of apoptosis or necrosis (Kroemer *et al*, 2007). Although the role of pro-apoptotic proteins, BAX and BAK, in this process is clear, the nature of how these are activated to initiate MOMP is currently under debate. In particular, how BH3-only BCL-2 family proteins initiate BAX/BAK-assisted mitochondrial permeability pore formation remains unclear. It has been suggested that a subset of BH3-only proteins act as sensitisers to the action of other BH3-only proteins thought to play the role of BAX/BAK activators (Willis and Adams, 2005). Other recent evidences suggest that BH3-only proteins, such as NOXA, BAD and BIK/NBK, may act as inactivators of antiapoptotic BCL-2/BCL-XL and MCL-1, preventing their inhibitory interaction with activator BH3-only proteins such as PUMA, BID and BIM (Kim *et al*, 2006). In either event, it is clear that the increased activity of pro-apoptotic BH3-only proteins is important for the initiation of cell death by various stimuli.

Inositol hexakisphosphate (IP6) is a naturally occurring phytochemical abundant in soy and legumes that exhibits anticancer activity in a wide range of cancers (Vucenik and Shamsuddin, 2003). Although definite mechanisms have yet to be established, IP6 activity has been reported to involve several processes (Fox and Eberl, 2002). In DU145 AIPCa cells, IP6 has been suggested to inhibit phosphatidyl inositol-3-kinase, prevent epidermal growth factor receptor signalling through the mitogen-activated protein kinase signalling cascade (Zi *et al*, 2000) and diminish constitutive NF-*κ*B activity (Agarwal *et al*, 2003). Still, in the context of DU145 cells, IP6 was found to modulate cdk–cyclin and pRb/E2F complexes leading to p21 and p27 upregulation and cell cycle arrest in G1 (Singh *et al*, 2003).

Treatment of AIPCa cells with IP6 can also induce classic hallmarks of apoptotic death such as caspase-3 activation, cleavage of poly ADP-ribose polymerase, increased cell-surface phosphatidylserine and DNA fragmentation (Singh *et al*, 2003; Diallo *et al*, 2006). In LNCaP cells, treatment with IP6 was found to increase the expression of pro-apoptotic BAX while decreasing the levels of antiapoptotic BCL-2 (Agarwal *et al*, 2004). In addition, we have recently shown that in PC3 cells, IP6 induces the mRNA expression of *PUMA*, *NOXA* and *BAX* as well as a subset of NF-*κ*B-responsive genes including *IκB-α* and *IRF-2* (Diallo *et al*, 2006). As such, we hypothesised that the upregulation of NF-*κ*B-responsive genes as well as of genes coding for pro-apoptotic proteins could play a role in mediating the pro-apoptotic effects of IP6 in PC3 cells. In this study, we investigated the role of NF-*κ*B and BCL-2 family members in IP6-induced cell death. We also evaluated whether IP6 could be useful in combination with proteasome inhibitors.

## Materials and methods

### Cell culture

PC3 and DU145 cell lines were obtained from ATCC (Manassas, VA, USA). Cells were maintained in RPMI-1640 complemented with 10% foetal calf serum, gentamicin (50 *μ*g ml^−1^) and amphotericin B (250 ng ml^−1^) (Gibco-BRL, Frederick, MD, USA). Cell cultures were incubated at 37°C in a humidified 5% CO_2_ atmosphere and subcultured at 1 : 3 (LNCaP) or 1 : 5 (PC3, 22Rv1, DU145) by trypsinisation with 0.25% trypsin for 5–10 min at 37°C (Gibco-BRL).

### Drugs and inhibitors

Inositol hexakisphosphate *(Myo*-Inositol hexakisphosphate dodecasodium salt; Sigma-Aldrich, St Louis, MO, USA) was kept as a 100 mM stock solution diluted in water. Actinomycin D (Sigma-Aldrich) was dissolved in DMSO and kept as a 1 mg ml^−1^ stock solution. Cycloheximide (Supelco, Bellefonte, PA, USA) stock solution (50 mg ml^−1^) was dissolved in ethanol. MG-132 (Calbiochem, San Diego, CA, USA) was also diluted in ethanol as a 20 mM stock solution. ALLN (Calbiochem) was reconstituted in DMSO and kept at a stock concentration of 10 mM.

### Cell seeding and treatments

For WST-1 assays, whole-cell extracts, nuclear/cytoplasmic extracts, RNA extractions and for 5,5′,6,6′-tetrachloro-1,1′,3,3′-tetraethyl-benzimidazolcarbocyanide iodide (JC-1) assays, a similar cell-seeding procedure was used. Briefly, cells were trypsinised and counted on a haemocytometer, then diluted in the appropriate media at 200 000 cells per ml and distributed in 96-well plates (100 *μ*l per well for WST-1 and luciferase reporter assays) or six-well plates (2 ml per well for JC-1 assay and RNA extractions). For whole-cell and nuclear/cytoplasmic extracts, cells (at a density of 200 000 cells per ml) were seeded in 60 mm petris (3 ml per petri), 100 mm petris (5 ml per petri) or 150 mm petris (10 ml per petri). Cells were allowed to adhere overnight before treatment. For the experiments assessing the effects of dominant-negative (DN) I*κ*B-*α* on IP6 efficacy, cells were transfected with DN-I*κ*B-*α* (pCMV-I*κ*B-*α*M; Clontech, Palo Alto, CA, USA) or control plasmid pCMV-Neo (Clontech) 24 h before seeding. Efficacy of DN-I*κ*B-*α* transfection was verified in parallel by luciferase assay (see below). Cells were then treated with the indicated concentrations of IP6. In the experiments where the effect of actinomycin D, cycloheximide, MG-132, cycloheximide+MG-132 and ALLN on the activity of IP6 was assessed, cells were pre-treated 4 h before the addition of IP6.

### WST-1 metabolic assay

After a 24-h treatment with IP6 (in addition to treatment with the appropriate inhibitors where indicated), 10 *μ*l of WST-1 reagent (Roche, Nutley, NJ, USA) was added to wells and plates were incubated at 37°C in a humidified 5% CO_2_ atmosphere. Incubation times were optimised for each cell line as recommended by the manufacturer. WST-1 signal was measured on a Bio-Rad Model 3550 microplate reader at 450 nm with reference at a wavelength of 655 nm. Following data acquisition, cell metabolic activity was calculated by first subtracting the readout of the WST-1 background (media+WST-1) from all values. For each independent experiment, the median of the replicates was calculated for each treatment. Subsequently, relative metabolic activity was calculated as being the median WST-1 readout of the drug-treated well/median WST-1 readout of vehicle-treated well. In the cases where cells were pre-treated with an inhibitor (actinomycin D, cycloheximide or MG-132), values were normalised relative to the inhibitor-treated well that was not challenged with IP6. Cellular metabolic activity was plotted as a function of IP6 concentration.

### Protein extracts

Cells were scraped in their media, collected by centrifugation and washed twice with cold PBS. Pellets were frozen at −80°C. Subsequently, whole-cell extractions were performed by applying cold lysis buffer (10 mM Tris-Hcl (pH 7.4), 150 mM NaCl, 1 mM EDTA, 1 mM DTT, 1 mM NaF, 0.5% NP-40, 10 *μ*g ml^−1^ aprotinin, 2 *μ*g ml^−1^ leupeptin, 2 *μ*g ml^−1^ pepstatin, 10 *μ*mol l^−1^ phenylmethylsulphonyl fluoride and 200 *μ*mol l^−1^ orthovanadate) on ice for 30 min. Whole-cell extracts were collected after centrifugation in a Heraeus Biofuge (13 000 r.p.m. for 10 min at 4°C) and were immediately stored at −80°C. Protein concentration was measured by Bradford assays (Bio-Rad Laboratories Inc., Hercules, CA, USA) according to the manufacturer's instructions.

### Nuclear and cytoplasmic protein extracts

After cell treatments, media were aspirated, cells were scraped and washed twice with cold PBS, and pellets were frozen at −80°C. Ice-cold buffer I (10 mmol l^−1^ Hepes, 50 mmol l^−1^ NaCl, 10 mmol l^−1^ EDTA and 5 mmol l^−1^ MgCl_2_) with freshly added protease and phosphatase inhibitors (10 *μ*g ml^−1^ aprotinin, 2 *μ*g ml^−1^ leupeptin, 2 *μ*g ml^−1^ pepstatin, 10 *μ*mol l^−1^ phenylmethylsulphonyl fluoride and 200 *μ*mol l^−1^ orthovanadate) was added and cells were incubated on ice for 30 min. Cell membranes were lysed by incubating with 1% NP-40 for 10 min. Cytosolic fractions were collected after centrifugation (3000 **g** for 5 min at 4°C). Ice-cold buffer II (10 mmol l^−1^ Hepes, 400 mmol l^−1^ NaCl, 0.1 mmol l^−1^ EDTA, 0.5 mmol l^−1^ and DTT) with freshly added protease and phosphatase inhibitors was then added to the nuclear aggregates and incubated on ice for 1 h. Nuclear protein fractions were collected after centrifugation (14 000 **g** for 15 min at 4°C). Each fraction was immediately stored at −80°C.

### Antibodies

Antibodies recognising NOXA (OP180) and PUMA (PC686) were obtained from Calbiochem. Antibodies detecting BAX (N-20, sc-493), BCL-2 (C-2, sc-7382), BIK/NBK (N-19, sc-1710), MCL-1 (H-260, sc-20679), RAN (C-20, sc-1156), I*κ*B-*α* (C-21, sc-371), NF-*κ*B p65 (F-6, sc-8008), *α*-tubulin (TU-02, sc-8035), as well as horseradish peroxidase-conjugated secondary antibodies were obtained from Santa Cruz Biotechnology (Santa Cruz, CA, USA). The anti-NF-*κ*B p50 (06-886) antibody was purchased from Upstate (Lake Placid, NY, USA). Antibodies recognising *β*-actin (AC-15, ab6276) and GAPDH (ab-9485) were obtained from Abcam (Cambridge, MA, USA).

### Western blot analysis

For western blot analyses, 20–75 *μ*g of whole-cell protein or nuclear/cytoplasmic protein extracts were resolved on 10–17% polyacrylamide gels and then transferred onto nitrocellulose or PVDF membranes at 60 V for 1–2 h. Blots were blocked using 5% non-fat dry milk in PBS–Tween 0.1% buffer for 2 h at room temperature. Membranes were then probed using antibodies recognising PUMA (1 : 1000), NOXA (1 : 200), BAX (1 : 1000), MCL-1 (1 : 100), BIK/NBK (1 : 200), BCL-2 (1 : 200), RAN (1 : 3000), I*κ*B-*α* (1 : 1000), NF-*κ*B p65 (1 : 750), NF-*κ*B p50 (1 : 750), *α*-tubulin (1 : 1000), *β*-actin (1 : 10000) and GAPDH (1 : 5000). Membranes were then incubated with appropriate secondary antibodies conjugated to horseradish peroxidase (Amersham Life Sciences Inc., Arlington Heights, IL, USA) in blocking buffer for 45 min at room temperature and developed with enhanced chemiluminescence (ECL) substrate (Amersham Life Sciences Inc.). When necessary, membranes were stripped using the protocol described in the ECL kit (Amersham Life Sciences Inc.) and re-probed. Densometric analysis was performed using the BioRad Quantity One® software (CA, USA).

### Quantitative real-time PCR

After the indicated times of treatment with IP6 and/or actinomycin D, media were removed and RNA was extracted with Trizol reagent according to the manufacturers' instructions (Invitrogen, Burlington, ON, Canada). Concentration of RNA samples was determined using a Beckman DU-600 spectrophotometer. RNA (2 *μ*g) was used to synthesise a cDNA using the SuperScript first-strand synthesis system (random hexamer method) according to the manufacturer's instructions (Invitrogen). The QuantiTect SYBR Green PCR kit was used as recommended (Qiagen, Mississauga, ON, Canada). Real-time PCRs were performed on a Rotor-gene RG-300 (Corbett Research, Sydney, Australia). Optimal threshold and reaction efficiency were determined using the Rotor-gene software. Melt curves for each primer exhibited a single peak, indicating specific amplification, which was also confirmed by agarose gel. *C*_t_ values were determined using the Rotor-gene software at the optimal threshold previously determined for each primer. Relative I*κ*B-*α*/actin B ratios were calculated using the Pfaffl method (Pfaffl, 2001). Fold induction was calculated relative to the vehicle-treated control. Experiments were performed twice and real-time measurements were carried out in duplicate. Primer sequences used were described earlier (Diallo *et al*, 2006).

### JC-1 mitochondrial depolarisation assay

PC3 cells were incubated with 10 *μ*g ml^−1^ of JC-1 (Molecular Probes, Eugene, OR, USA) at 37°C in a humidified 5% CO_2_ atmosphere for 15 min. Cells were washed twice with PBS and trypsinised with 500 *μ*l 0.25% trypsin for 10 min. Cells were then collected and put in fluorescence-activated cell sorting (FACS) counting tubes containing 500 *μ*l RPMI-1640 complemented with 10% FCS. JC-1 emission was measured in individual cells using a Coulter Epics® XL-MCL. The fold change in cells presenting de-polarised mitochondria (green shift in JC-1 fluorescence) was calculated relative to vehicle-treated control for each time point.

### Luciferase reporter assays

PC3 cells were seeded at 400 000 cells per ml in 60 mm petris (3 ml). The following morning, lipofectamine PLUS reagent (Invitrogen) was used to transfect 1 *μ*g pCMV-I*κ*B-*α*M (or pCMV-Neo control), 0.8 *μ*g of a 3enh-*κ*B-ConA-luc green luciferase reporter (or control ptkGL3 luciferase gene driven by a minimal thymidine kinase promoter) and 0.2 *μ*g of a pCMV-RL Renilla luciferase reporter (Promega, Madison, WI, USA). The following day, transfected cells were re-seeded at a density of 20 000 cells per well in 96-well plates. To assess the effects of MG-132 on *κ*B-luc activity, transfected cells were then treated with MG-132 for 24 h after which time green and renilla luciferase activity was assayed using the dual luciferase reporter assay system (Promega) on a Victor^3^ luminometer/fluorimeter (Perkin Elmer, ON, Canada). To calculate the relative *κ*B reporter activity, green luciferase activity was first normalised according to renilla luciferase output (transfection efficacy control) and then corrected for background effects by subtracting the basal activity detected from the ptkGL3 plasmid. The 3enh-*κ*B-ConA-luc (hereby referred to as *κ*B-luc), carrying a firefly luciferase gene under the control of a trimer of *κ*B consensus, was a kind gift from Dr Juana Wietzerbin (Institut Curie, Paris, France). To construct the ptkGL3 control plasmid, the SV40 promoter (*Bgl*II/*Hin*dII fragment) was removed from the pGL3 basic plasmid (Promega) and a minimal thymidine kinase promoter (*Bgl*II/*Hin*dIII fragment) from the pNF-*κ*B-d2EGFP plasmid (Clontech) was inserted in its place.

### Statistics

Averages, standard errors of the mean and *P*-values (calculated using *t*-test) were calculated from individual experiments. The number of replicates as well as the number of independent experiments are indicated in the figure legends. All statistical tests were performed in Excel (Microsoft®, WA, USA). For WST-1 assays, an effect was deemed significant when a *P*-value <0.05 was reached for at least one concentration of IP6.

## Results

### IP6 induces NF-*κ*B nuclear translocation in PC3 cells

**Figure 1:**
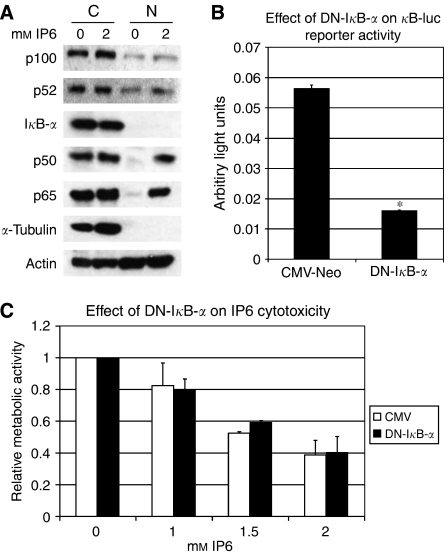
(**A**) IP6 induces canonical NF-*κ*B subunit nuclear translocation. Nuclear (N) and cytoplasmic (C) extracts of PC3 cells, treated with vehicle (0 mM) or IP6 (2 mM) for 24 h, were probed by western blot to assess the expression and subcellular localisation of p100, p52, I*κ*B-*α*, p50 and p65. *α*-Tubulin was used as a control for nuclear extract purity. *β*-Actin was used as a loading control. (**B**) A non-degradable dominant-negative form of I*κ*B-*α* inhibits NF-*κ*B transcriptional activity. DN-I*κ*B-*α* or pCMVNeo control plasmids were co-transfected with *κ*B-luc/Renilla luciferase in PC3 cells. *κ*B-luc activity was measured after 72 h and normalised according to renilla and basal tkGL3 activity (see Materials and Methods). Data represent average of two independent experiments performed in four to eight replicates each. ^*^P-value <0.05. (**C**) Inhibition of NF-*κ*B transcriptional activity does not modulate the efficacy of IP6. PC3 cells, transiently transfected (48 h) with pCMVNeo and DN-I*κ*B-*α* cells, were plated at a density of 20 000 cells per well and treated with increasing doses of IP6. Metabolic activity was measured using WST-1 reagent and relative metabolic activity was normalised according to vehicle-treated pCMVNeo- or DN-I*κ*B-*α*-transfected cells (see Materials and Methods). Data represent average of two independent experiments performed in three to six replicates each. Error bars represent the standard error.

We have previously observed that a subset of NF-*κ*B-responsive genes is upregulated in response to IP6 in PC3 cells. Notably, we have found that I*κ*B*-α* mRNA levels can increase up to 20-fold following a 24-h treatment with 2 mM IP6 (Diallo *et al*, 2006), beginning as early as 4 h post-treatment (data not shown). As I*κ*B-*α* is thought to be regulated by p50/p65 NF-*κ*B subunits (Sun *et al*, 1993), we set out to determine the effects of IP6 on NF-*κ*B subunit nuclear translocation. Figure 1A shows that although only minimal changes in the status of p52 and its precursor p100 could be observed, nuclear p50 and p65 increased substantially following a 24 h treatment with IP6 (Figure 1A), an event that was visible as early as 4 h following challenge with IP6 (data not shown). Notably, the increase in nuclear p50/p65 was not due to contamination from cytoplasmic proteins, as evidenced by the absence of *α*-tubulin in the nuclear extracts (Figure 1A). In contrast to observations at the mRNA level (Diallo *et al*, 2006), a slight decrease in I*κ*B-*α* protein (which was primarily located in the cytoplasm) was detected in parallel (Figures 1A and 3). Overall, these data indicated that canonical NF-*κ*B subunits translocate to the nucleus in response to treatment with IP6.

### Reduced NF-*κ*B transcriptional activity by expression of a non-degradable I*κ*B-*α* does not modulate the response to IP6

Others have reported that in DU145 cells, NF-*κ*B nuclear translocation decreases in response to IP6, resulting in a reduced NF-*κ*B DNA-binding activity (Agarwal *et al*, 2003). Consequently, the observation that NF-*κ*B p50 and p65 translocate to the nucleus in response to IP6 in PC3 cells led us to ask whether the modulation of NF-*κ*B-mediated transcription was implicated in the activity of IP6 in PC3 cells. To address this, we transfected PC3 cells with a non-degradable DN mutant of I*κ*B-*α* (Brown *et al*, 1995) (DN-I*κ*B-*α*) or a control vector (pCMVNeo) and determined whether this could modulate the effects of IP6. As shown in Figure 1B, the transient transfection of DN-I*κ*B-*α* effectively reduced NF-*κ*B transcriptional activity relative to the pCMVNeo control as assessed using the *κ*B-luc reporter. However, WST-1 assays indicate that DN-I*κ*B-*α-* and pCMVNeo-transfected PC3 cells respond similarly to a challenge with IP6 (Figure 1C). While not excluding any potential effects of reduced NF-B activity on cell proliferation (Diallo *et al*, 2006), this suggests that reduced NF-*κ*B transcriptional activity does not protect from or enhance the cytotoxic effects of IP6.

### IP6 modulates BCL-2 family protein expression

**Figure 2:**
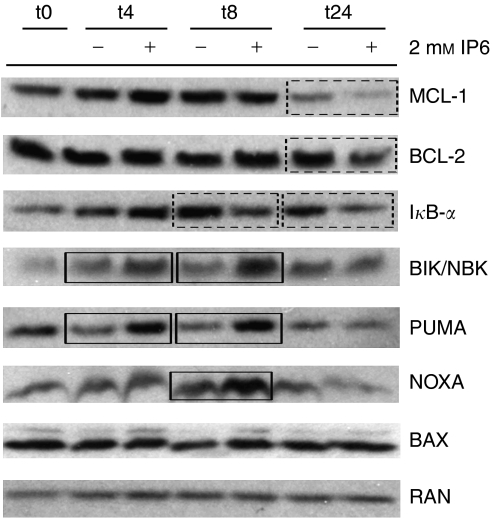
IP6 modulates the expression of BCL-2 family proteins. PC3 cells were treated with 2 mM IP6 or vehicle and whole-cell extracts were prepared from cells harvested upon treatment (t0) and after 4 h (t4) and 8 h (t8) of additional treatment with IP6 (+) or vehicle (−). Western blotting was used to probe extracts for MCL-1, BCL-2, I*κ*B-*α*, BIK/NBK, PUMA, NOXA and BAX expression. RAN expression was used as a loading control. Solid boxes signal increased protein levels in treated cells as compared with time-matched control cells, whereas dashed boxes indicate decreased protein levels relative to time-matched controls. Blot was representative of two independent experiments each performed in duplicate.

In parallel with NF-*κ*B-responsive genes, we have previously reported that the mRNA levels of BCL-2 family pro-apoptotic genes such as *NOXA*, *PUMA* and *BAX* increase in response to IP6 (Diallo *et al*, 2006). Hence, we next set out to determine how the protein levels of these pro-apoptotic BCL-2 family proteins changed in time following treatment with IP6. To address this, PC3 cells were challenged with 2 mM IP6, and whole-cell extracts were prepared after 4, 8 and 24 h of continuous treatment. The extracts were then probed by western blot. As shown in Figure 2, the expression of PUMA increased as early as 4 h following treatment with IP6 until at least 8 h post-treatment. Similarly, an increased NOXA expression was also observed although later (8 h) and to a lesser extent. Both PUMA and NOXA expression subsequently returned to normal after 24 h of treatment with IP6. In contrast, no clear changes in BAX protein expression were observed. We also looked at whether protein expression of other pro- and antiapoptotic BCL-2 family members could be modulated in response to IP6. As can be seen in Figure 2, BIK/NBK expression is also upregulated by IP6 similarly to PUMA though to a lesser extent, particularly at 4 h. In contrast, we could observe a decreased expression of antiapoptotic proteins MCL-1 and BCL-2, though only after 24 h of treatment with IP6. These data suggested that IP6 induces temporal changes in the ratio of pro- and antiapoptotic BCL-2 family proteins.

### Protein synthesis is important for IP6-induced cytotoxicity

**Figure 3:**
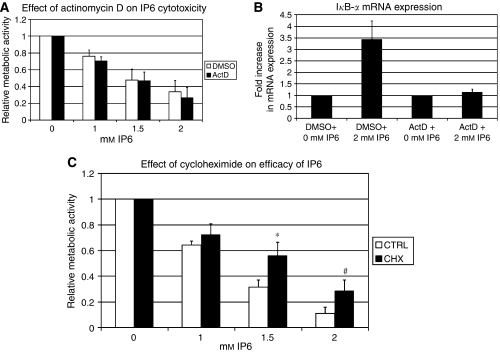
(**A**) A transcription inhibitor does not protect PC3 cells from the effects of IP6. Actinomycin D (1 *μ*g ml^−1^)- and DMSO-pre-treated PC3 cells (density of 20 000 cells per well) were treated with increasing doses of IP6 for 24 h. Metabolic activity was measured after 24 h (plus 4-h pre-treatment) using WST-1, and the data were normalised according to DMSO- or actinomycin D-pre-treated cells not treated with IP6. Data represent average of five independent experiments each performed in three to six replicates. No significant difference was observed between actinomycin D- and DMSO-treated cells. (**B**) Actinomycin D inhibits IP6-induced upregulation of I*κ*B-*α* mRNA. PC3 cells were pre-treated for 4 h with 1 *μ*g ml^−1^ actinomycin D or DMSO, then treated with 2 mM IP6 or vehicle (0 mM). cDNA was synthesised from RNA extracted 24 h later and real-time PCR was used to measure the fold change in I*κ*B-*α* mRNA expression. Data represent an average of two independent experiments performed in duplicate. (**C**) An inhibitor of protein translation protects PC3 cells from the effects of IP6. As in (**B**), cycloheximide (50 *μ*g ml^−1^)- and ethanol-pre-treated PC3 cells were treated with increasing doses of IP6 for 24 h and metabolic activity was measured using WST-1. Data represent an average of three independent experiments performed in three to six replicates. Error bars represent the standard error. ^*^P-value <0.05; ^#^*P*-value=0.06.

In light of the results presented in Figure 2, in addition to our observation of increased mRNA expression of pro-apoptotic BCL-2 family members in a previous study (Diallo *et al*, 2006), we next set out to determine whether transcriptional upregulation of pro-apoptotic genes, such as *PUMA*, *NOXA* and potentially *BIK/NBK*, could be important for mediating the effects of IP6. To gain an overall appreciation for this, we compared the efficacy of IP6 in PC3 cells pre-treated (4h) with an inhibitor of mRNA transcription (Yamamoto *et al*, 1980) or vehicle (DMSO). Figure 3A shows that continuous treatment with actinomycin D, starting 4 h before the addition of IP6 (for 24 h), did not modulate PC3 cell sensitivity to IP6. To verify that actinomycin D effectively blocked mRNA synthesis in our conditions, we looked at the mRNA levels of I*κ*B-*α* in response to IP6. Figure 3B shows that IP6-induced I*κ*B-*α* upregulation after 24 h of treatment was effectively blocked by actinomycin D, but not by DMSO. As IP6 treatment could lead to protein upregulation independently of transcription, we next assessed the efficacy of IP6 in PC3 cells where protein production was blocked using cycloheximide (50 *μ*g ml^−1^, 4 h pre-treatment). Figure 3C shows that a continuous treatment with the translation inhibitor cycloheximide (Yamamoto *et al*, 1980), starting 4 h before treatment with IP6, significantly protected from IP6-induced toxicity (*P*<0.05 at 1.5 mM IP6). These data suggested a role for protein synthesis in mediating the effects of IP6 and that one possible effect of blocking protein translation would be to hinder on the temporal shift of the ratio between pro-apoptotic to antiapoptotic proteins induced by IP6.

### A proteasome inhibitor sensitises androgen-independent prostate cancer cells to the effects of IP6

**Figure 4:**
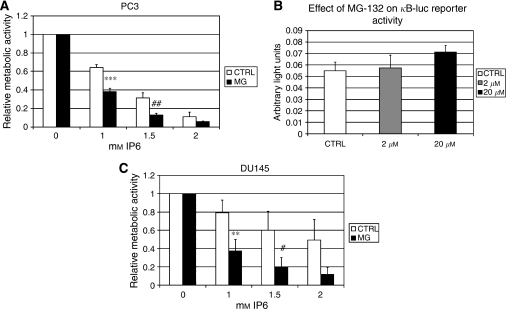
The proteasome inhibitor MG-132 sensitises AIPCa cells to the effects of IP6. In (**A**) (PC3) and (**C**) (DU145), cells were plated at 20 000 cells per well and pre-treated for 4 h with MG-132 (or ethanol control) before addition of IP6 at increasing concentrations. Metabolic activity was measured 24 h later using WST-1. For each cell line, relative metabolic activity was calculated according to the WST-1 output MG-132-treated or control cells treated with vehicle (0 mM IP6). Data represent an average of three independent experiments performed in three to six replicates each. ^***^P-value<0.005; ^**^P-value<0.01; ^#^*P*-value=0.08; ^##^*P*=0.09. (**B**) As described in Figure 1C, *κ*B-luc reporter and control-transfected cells were treated with 2 or 20 *μ*M MG-132 or vehicle (CTRL) and assayed for relative luciferase activity. Data represent an average of two independent experiments performed in four to eight replicates each. Error bars represent the standard error.

As blocking protein translation reduced the efficacy of IP6, potentially by preventing increases in pro-apoptotic protein levels, we rationalised that blocking proteasome-mediated protein degradation could have the opposite effect and instead enhance the effect of IP6. To test this hypothesis, we treated PC3 AIPCa cells with the commonly used proteasome inhibitor MG-132 (20 *μ*M) (Palombella *et al*, 1994), beginning 4 h before the addition of IP6, and assessed the efficacy of IP6 using WST-1 assays. Figure 4A shows that over 24 h, cells co-treated with MG-132 were significantly more sensitive to IP6 treatment than control cells (*P*<0.005, IP6 1 mM). As inhibitors of the proteasome have been reported to inhibit NF-*κ*B transcriptional activity, we also assessed the effect of MG-132 on *κ*B-luc reporter activity in PC3 cells. We found that, at least in the PC3 cell line, MG-132 did not inhibit NF-*κ*B-mediated transcription of the *κ*B-luc reporter. In fact, a slight increase in *κ*B-luc reporter activity was detected with increasing doses of MG-132 (Figure 4B). MG-132 co-treatment also significantly sensitised DU145 AIPCa cells to the effects of IP6 (Figure 4C; *P*<0.01, IP6 1 mM).

### IP6 and proteasome inhibitors elicit enhanced mitochondrial depolarisation in a protein translation-dependent fashion

**Figure 5:**
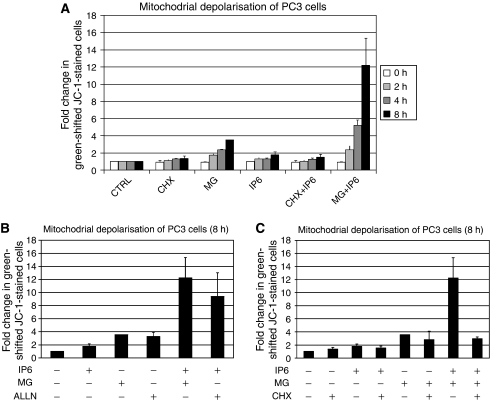
IP6 and proteasome inhibitors present an enhanced effect on mitochondrial depolarisation. (**A**) PC3 cells were pre-treated with 50 *μ*g ml^−1^ cycloheximide (CHX), 20 *μ*M MG-132 (MG) or control ethanol and the percentage of cells presenting mitochondrial depolarisation according to JC-1 staining was assessed by FACS after 0, 2, 4 and 8 h of treatment with 2 mM IP6 (or vehicle). (**B**) PC3 cells were pre-treated with 20 *μ*M MG-132, 10 *μ*M ALLN or DMSO before the addition of 2 mM IP6 (or control). Eight hours later, the percentage of cells presenting mitochondrial depolarisation according to JC-1 staining was assessed by FACS. (**C**) PC3 cells were pre-treated with 20 *μ*M MG-132, 50 *μ*g ml^−1^ cycloheximide or a combination of MG-132 and cycloheximide or control ethanol before addition of 2 mM IP6 (or vehicle). Eight hours later, the percentage of cells presenting mitochondrial depolarisation according to JC-1 staining was assessed by FACS. In (**A**–**C**), data were normalised according to the percentage of cells exhibiting mitochondrial depolarisation according to the JC-1 staining observed in vehicle-treated controls. Data represent an average of two to three independent experiments. All error bars represent the standard error.

The potential involvement of one or more members of the BCL-2 family proteins in the observed effect of IP6 next prompted us to look at whether a treatment with IP6 and/or proteasome inhibitors initiated MOMP. To address this, we stained both treated and control PC3 cells with JC-1 dye and used FACS to measure the percentage of cells exhibiting a green shift in JC-1 fluorescence in treated cells relative to the control at various time points following treatment. Notably, a JC-1 fluorescence shift from red to green is indicative of cells having undergone MOMP (Salvioli *et al*, 1997). We found that IP6 (2 mM) used alone caused a modest but time-dependent increase in MOMP (Figure 5A–C). As we had observed that cycloheximide protected from the effects of IP6 using WST-1 assays, we looked at whether cycloheximide (50 *μ*g ml^−1^) could protect from IP6-induced MOMP. We found that cycloheximide on its own could induce MOMP to an extent similar to what was observed for IP6 (Figure 5A and C). However, co-treatment with IP6 and cycloheximide did not further increase the amount of cells exhibiting a green shift in JC-1 fluorescence (Figure 5A and C). Similarly, we found that MG-132 used alone also caused a time-dependent increase in green-shifted JC-1-stained cells. Although likely due to the extra 4 h of pre-treatment with MG-132, at 8 h (plus 4 h ‘pre-treatment’), MG-132 appeared to induce roughly twice the relative amount of MOMP as compared with IP6 (Figure 5A–C). Importantly, co-treatment with MG-132 and IP6 caused a drastic increase in cells presenting MOMP. After 8 h, cells co-treated with IP6 and MG-132 had nearly three-fold the quantity of cells exhibiting MOMP as compared with MG-132 alone and six-fold the quantity of cells exhibiting MOMP as compared with IP6-treated cells (Figure 5A–C). We next questioned whether the observed enhanced effect of combined IP6 and MG-132 at the level of MOMP was specific to MG-132 or whether it could also be observed with other proteasome inhibitors. Figure 5B shows that ALLN (10 *μ*M) (Schow and Joly, 1997) also enhances the induction of MOMP when combined with IP6 in PC3 cells. To assess whether the enhancement of MOMP, elicited by combined treatment with IP6 and proteasome inhibitors, required *de novo* protein synthesis, we co-treated cells with cycloheximide (50 *μ*g ml^−1^), MG-132 (20 *μ*M) and IP6 (2 mM). We found that although the addition of cycloheximide did not reduce MG-132-induced MOMP (Figure 5A and C), the addition of cycloheximide almost completely blocked the enhanced MOMP observed in response to combined treatment with MG-132 and IP6 (Figure 5C).

### MG-132 and cycloheximide modulate the expression of BCL-2 family proteins in PC3 cells

**Figure 6:**
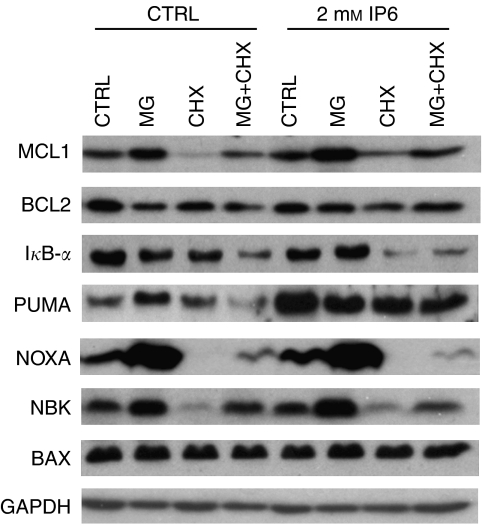
Modulation of BCL-2 family protein expression in PC3 cells in response to IP6. MG-132 (MG) and cycloheximide (CHX). PC3 cells were pre-treated for 4 h with 20 *μ*M MG-132, 50 *μ*g ml^−1^ cycloheximide or both. Cells were then challenged with 2 mM IP6 (or control) for 4 h after which whole-cell extracts were prepared and probed for MCL-1, BCL-2, I*κ*B-*α*, PUMA, NOXA, BIK/NBK and BAX by western blot. GAPDH was used as a loading control. The presented blots were representative of three independent experiments each performed in duplicate.

As blocking of protein translation prevented MOMP in response to combined IP6 and MG-132 treatment, we deemed it relevant to look at how BCL-2 family member expression was modulated in response to MG-132 and cycloheximide with and without IP6 (added 4 h later). Western blotting of whole-cell extracts was used to detect the expression of BCL-2 family members as well as I*κ*B-*α*. The results presented in Figure 6 suggest that treatment of PC3 cells with MG-132 substantially increased the levels of NOXA and, to a lesser extent, of BIK/NBK, PUMA and MCL-1. The addition of IP6 led to a slight further increase in the levels of NOXA, BIK/NBK and MCL-1. In contrast, the treatment of PC3 cells with cycloheximide on its own caused almost a complete loss of NOXA expression and substantially decreased BIK/NBK and MCL-1 protein levels. Interestingly, subsequent addition of IP6 curtailed the decrease in the levels of MCL-1 but not that of NOXA or BIK/NBK. Cycloheximide did not modulate the expression of PUMA or BAX when used alone although, notably, it did not completely block IP6-induced upregulation of PUMA. Similar to MG-132, cycloheximide used alone slightly decreased the expression of I*κ*B-*α*. However, the addition of IP6 to cycloheximide-treated cells caused a further decrease in I*κ*B-*α*. Although co-treating PC3 cells with MG-132 and cycloheximide nullified their respective effects on MCL-1 and BIK/NBK expression levels, reduced NOXA and BCL-2 expression remained observable. Interestingly, the combined treatment with MG-132 and cycloheximide reduced I*κ*B-*α* levels more than either treatment used alone, independently of IP6.

## Discussion

In contrast with studies performed using other cell lines (Ferry *et al*, 2002; Agarwal *et al*, 2003), the data presented here suggest that, at least in PC3 cells, classical NF-*κ*B subunits p50 and p65 translocate to the nucleus up to 24 h following treatment with IP6 (Figure 1A). In parallel, I*κ*B-*α* protein levels slightly decrease in response to IP6 (Figures 1A and 2), even though I*κ*B-*α* mRNA increases substantially over 24 h (Diallo *et al*, 2006), beginning as early as 4 h post-treatment (data not shown). Although the I*κ*B-*α* gene is a well-known target of p50/p65, the I*κ*B-*α* protein is normally degraded upon activation of the classical pathway following phosphorylation by IKK-*β*, leading to an autofeedback loop (Karin and Ben-Neriah, 2000). Hence, our observations of nuclear translocation of p50/p65, in parallel with decreased I*κ*B-*α* protein and concomitant increase of I*κ*B-*α* mRNA, are consistent with the activation of the classical NF-*κ*B pathway. This is further reinforced by the observation that blocking protein production using cycloheximide (which we expect would prevent any new I*κ*B-*α* from being produced) further decreased the expression of I*κ*B-*α* protein in response to IP6 (Figure 6). In contrast, we found that the non-canonical pathway (p100/p52) is largely unaffected by treatment with IP6. Although these results altogether suggest that the NF-*κ*B classical pathway may be triggered in response to treatment of PC3 cells with IP6, inhibiting NF-*κ*B transcriptional activity (assessed by *κ*B-luc assay, Figure 1B), by transfecting PC3 cells with a non-degradable DN form of I*κ*B-*α* (Brown *et al*, 1995), does not modulate PC3 cell sensitivity to IP6 (Figure 1C). Hence, our results are at odds with the suggestion that IP6-induced cell death is mediated by the inhibition of constitutive NF-*κ*B activity.

On the basis of our results, a more likely possibility is that IP6 modulates BCL-2 family protein expression in a manner that leads to an increased pro-apoptotic/antiapoptotic BCL-2 family protein ratio. Indeed, Figure 2 shows that IP6 induced a gradual shift in the levels of BCL-2 family members at the protein level, wherein pro-apoptotic proteins such as PUMA, NOXA and BIK were upregulated early on (4 and 8 h) and where the levels of antiapoptotic proteins such as MCL-1 and BCL-2 decreased somewhere between 8 and 24h following treatment with IP6. As we had previously observed the upregulation of *PUMA*, *NOXA* and *BAX* mRNA, we initially hypothesised that this could be mediated by the upregulation of pro-apoptotic genes at the mRNA level. However, we were surprised to find that a transcription inhibitor (Yamamoto *et al*, 1980), although effectively blocking IP6-induced upregulation of gene expression (Figure 3B), did not protect cells from the effects of IP6 (Figure 3A). As protein expression can be modulated by post-transcriptional events including mRNA export, mRNA translation and proteasome-mediated protein degradation, it remained possible that the ratio of pro-apoptotic to antiapoptotic proteins could shift in response to IP6 independently of mRNA transcription and actinomycin D. Indeed, PC3 cells in which protein translation was blocked using cycloheximide were significantly protected from the effects of IP6 (Figure 3C). At the very least, these data suggest that protein translation is more relevant than mRNA transcription in communicating the observed effects of IP6. With respect to this, it is a likely possibility that the effect of IP6 at least partially involves increased ratios of pro-apoptotic to antiapoptotic BCL-2 family proteins.

One potential mechanism that could lead to an increased protein production independently of transcription is that IP6 stabilises pro-apoptotic proteins. For example, this could result from the inhibition of specific ubiquitin ligases such as MULE/ARF-BP1 (that targets MCL-1 for degradation) (Zhong *et al*, 2005) or from the direct inhibition of the proteasome. On the other hand, the observed decrease in MCl-1, BCL-2 and I*κ*B-*α*, after longer exposure times of PC3 cells with IP6, argues against both of these possibilities (Figure 2). Another potential mechanism is that IP6 stimulates ribosomal protein production of pro-apoptotic proteins. Potential candidate targets that could mediate such an effect of IP6 include mTOR and RalA, which have been recently found to regulate the translation of the antiapoptotic protein FLIP_S_ (Panner *et al*, 2006). Notably, mTOR is well known to be regulated by Akt (Dutcher, 2004), which in turn is also an upstream activator of NF-*κ*B (Karin and Ben-Neriah, 2000; Karin *et al*, 2002; Chen and Greene, 2004), whose classical p50/p65 subunits translocate to the nucleus in response to IP6 (Figure 1A). However, the activation of mTOR is generally associated with an increased expression of cyclin D1 and cell proliferation, not cell death (Dutcher, 2004). Finally, reports showing that endogenous IP6 plays a role in mRNA export (Alcazar-Roman *et al*, 2006; Weirich *et al*, 2006) suggest the intriguing possibility that IP6 increases the cytoplasmic export of existing pools of accumulated pro-apoptotic nuclear mRNA. However, it is unclear whether the nuclear mRNA export machinery is so specific as to allow for the export of pro-apoptotic, but not antiapoptotic, BCL-2 family members.

Although the observed effect of cycloheximide on the efficacy of IP6 and IP6/MG-132 co-treatment may involve *de novo* protein synthesis, we cannot exclude the possibility that these observations are linked with reductions in basal pro-apoptotic protein levels resulting from pre-treatment with cycloheximide. Indeed, we can observe decreased levels of NOXA and BIK/NBK in response to treatment with cycloheximide alone (Figure 6, lane 3). However, treatment with cycloheximide led to a similar decrease in antiapoptotic MCL-1 (Figure 6). Surprisingly, we found that MCL-1 levels decreased less in response to treatment with cycloheximide when cells were subsequently treated with IP6 (Figure 6, compare lane 3 with 7). Analogously, IP6-induced expression of PUMA was not fully inhibited in the presence of cycloheximide (Figure 6, compare lane 4 with 7). This observation is interesting in light of reports suggesting that cycloheximide inhibits cytoplasmic ribosomes but not mitochondrial ribosomes, which are of notably different composition (Bruce and Kerry, 1987). Although relatively little is known about nuclear mRNA sorting to the mitochondria (Sylvestre *et al*, 2003), mitochondrial import of nuclear tRNAs is better described and is required for mitochondrial protein translation (Verner, 1993; Kamenski *et al*, 2007). It is tempting to speculate that certain BCL-2 family proteins can be synthesised by mitochondrial ribosomes from imported cytoplasmic mRNAs.

Following from our observations in Figures 2 and 3, we rationalised that if blocking the production of pro-apoptotic BCL-2 family proteins by treatment with cycloheximide reduced IP6 efficacy, then increasing their levels using proteasome inhibitors may lead to an enhanced effect. Indeed, we found that co-treatment with MG-132 significantly increased the effect of IP6 in at least two AIPCa cell lines (Figure 4A and B). Our results suggest that this effect is not due to the inhibition of NF-*κ*B transcriptional activity as treatment of PC3 cells with MG-132 increased NF-*κ*B transcription from a *κ*B-luc reporter (Figure 4B) and reduced I*κ*B-*α* protein levels (Figure 6, lanes 4 and 8). This is consistent with other studies that have reported that at least in some cells, proteasome inhibitors do not inhibit NF-*κ*B but instead increase the expression of BIK/NBK, while levels of BAX and BAK remain stable (Zhu *et al*, 2005). Indeed, we found that MG-132 clearly increased the levels of NOXA, BIK/NBK and MCL-1, but levels of BAX remained stable. Interestingly, treatment of PC3 cells with cycloheximide led to decreased levels of NOXA, BIK/NBK and MCL-1, suggesting that these may be quickly synthesised and degraded equally rapidly by the proteasome. As MG-132 has been reported to mainly inhibit chymotryptic proteasome activity, this may also suggest that the degradation of I*κ*B-*α* in prostate cells could be mediated by peptidylglutamyl peptide-hydrolysing, trypsin-like, or even caspase-like, proteasome-associated activities (Lee and Goldberg, 1998; Groll *et al*, 1999). In contrast, NOXA, BIK/NBK and MCL-1 may be the preferred clients of the chymotryptic active site.

The observation that blocking protein translation using cycloheximide almost completely inhibits MOMP induced by combined IP6- and proteasome-inhibitor treatment strongly points to the involvement of pro-apoptotic BCL-2 family members. In particular, proteins with potentially rapid degradation kinetics, such as NOXA and BIK/NBK, which are further increased by treatment with IP6 (albeit only slightly), may act in concert to elicit this enhanced effect on mitochondrial depolarisation. At the same time, it must be noted that antiapoptotic MCL-1 also seems to respond, particularly, to combined MG-132 and IP6 treatment. With respect to this, it will be interesting to determine whether a MCL-1-targeting siRNA can further enhance the efficacy of combined IP6/proteasome inhibitor treatment. Notably, others were able to increase the potency of bortezomib using this MCL-1 knockdown approach in melanoma cells (Qin *et al*, 2006).

In light of the multitude of pro-apoptotic BCL-2 family members, it is likely that other BCL-2 family members are also upregulated by both IP6 and MG-132, further perturbing the pro-apoptotic/antiapoptotic ratio. It is equally possible that a subset of pro-apoptotic proteins is specifically upregulated by IP6, whereas another complementary subset is upregulated by MG-132. This could lead to an enhanced cytotoxicity according to a recently proposed hierarchical BCL-2 family model of BAX/BAK activation. In this model, some BH3-only proteins such as NOXA, BAD, BMF and BIK/NBK may act upstream of ‘activator-only’ pro-apoptotic proteins such as BID, BIM and PUMA, by releasing them from the inhibitory clutches of antiapoptotic BCL-2/BCL-XL and MCL-1 (Kim *et al*, 2006). In this context, the combined upregulation of NOXA and BIK/NBK by MG-132 may complement the upregulation of activator-only proteins following challenge with IP6. Although PUMA was a potential candidate, its expression also appears to be increased by MG-132 (Figure 6, lane 2). In fact, levels of PUMA in cells treated with the combination of IP6 and MG-132 appeared closer to that observed in cells treated with MG-132 alone (Figure 6, lane 6). These levels were notably lower than those seen when treating cells with IP6 alone (Figure 6, lane 5), perhaps suggesting that the effect of MG-132 on PUMA protein levels is dominant over that of IP6. In addition, our attempts to prevent the enhanced cytotoxic effects of combined IP6 and MG-132 treatment by shRNA-mediated downregulation of PUMA have proved unsuccessful so far (data not shown). Hence, the net level of PUMA protein is not a likely mediator of this combined effect. Rather, we suspect that one or more pro-apoptotic ‘activator-only’ proteins not probed for here may be upregulated by IP6 but not by MG-132, or that, alternately, IP6 induces post-translational modifications that activate pro-apoptotic proteins upregulated by MG-132. We are currently investigating these possibilities.

Nonetheless, the enhanced effect of IP6 was seen with two different proteasome inhibitors. This was most clearly visible when MOMP was assessed in individual PC3 cells using JC-1 dye (Figure 5A–C). In addition to the observation that combining IP6 with MG-132 leads to an enhanced effect in both PC3 and DU145 cell lines, these results suggest that IP6 may be useful in combination with proteasome inhibitor drugs for treatment of AIPCa. This possibility is particularly exciting as proteasome inhibitors are already undergoing pre-clinical trials for AIPCa (Papandreou and Logothetis, 2004; Papandreou *et al*, 2004; Price and Dreicer, 2004) and as previous studies have also shown that IP6 can exhibit anticancer activity in mice when administered as a dietary supplement in drinking water (Singh *et al*, 2004).

## Conclusion

In conclusion, we find that NF-*κ*B does not likely play a major role in either preventing or promoting the cytotoxic effects of IP6 in PC3 cells. We propose that the upregulation of pro-apoptotic BCL-2 family members at the protein level is likely involved in skewing the pro-apoptotic/antiapoptotic protein ratio and in mediating the cytotoxic effects of IP6. Finally, we conclude that proteasome inhibitors enhance the effect of IP6 on mitochondrial depolarisation, potentially involving multiple pro-apoptotic BCL-2 family members. Altogether, our results suggest that the pre-clinical evaluation of proteasome inhibitors in combination with IP6 is warranted and could provide an alternative for the treatment of AIPCa.
